# One-Step Encapsulation of *ortho*-Disulfides in Functionalized Zinc MOF. Enabling Metal–Organic Frameworks in Agriculture

**DOI:** 10.1021/acsami.0c21488

**Published:** 2021-02-12

**Authors:** Francisco J. R. Mejías, Susana Trasobares, Rosa M. Varela, José M. G. Molinillo, José J. Calvino, Francisco A. Macías

**Affiliations:** † Allelopathy Group, Department of Organic Chemistry, Institute of Biomolecules (INBIO), Campus CEIA3, School of Science, University of Cádiz, C/República Saharaui, 7, Puerto Real, 11510 Cádiz, Spain; ‡ Departamento de Ciencia de los Materiales e Ingeniería Metalúrgica y Química Inorgánica, Facultad de Ciencias, Universidad de Cádiz, C/República Saharaui, 7, Puerto Real, 11510 Cádiz, Spain

**Keywords:** MOF, herbicide, encapsulation, weed control, iDPC

## Abstract

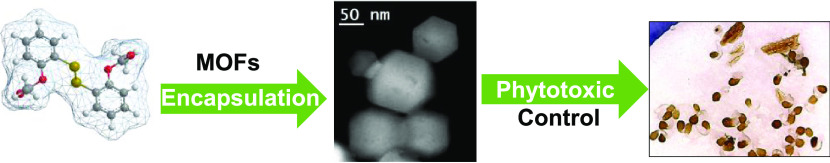

Application of natural products as new green agrochemicals with low average lifetime, low concentration doses, and safety is both complex and expensive due to chemical modification required to obtain desirable physicochemical properties. Transport, aqueous solubility, and bioavailability are some of the properties that have been improved using functionalized metal–organic frameworks based on zinc for the encapsulation of bioherbicides (*ortho*-disulfides). An *in situ* method has been applied to achieve encapsulation, which, in turn, led to an improvement in water solubility by more than 8 times after 2-hydroxypropyl-β-cyclodextrin HP-β-CD surface functionalization. High-resolution high-angle annular dark-field scanning transmission electron microscopy (HR HAADF-STEM) and integrated differential phase contrast (iDPC) imaging techniques were employed to verify the success of the encapsulation procedure and crystallinity of the sample. Inhibition studies on principal weeds that infect rice, corn, and potato crops gave results that exceed those obtained with the commercial herbicide Logran. This finding, along with a short synthesis period, *i*.*e*., 2 h at 25 °C, make the product an example of a new generation of natural-product-based herbicides with direct applications in agriculture.

## Introduction

Natural products are the main ecofriendly option for the protection and stimulation of crops all over the world.^1,2^ Nevertheless, the low water solubility of these compounds limits their broad application on a large scale. For this reason, green encapsulation has been investigated widely in recent years^3−5^ as a route to modulate the physicochemical properties of agrochemicals by changing the encapsulation agent.

In this respect, metal–organic frameworks (MOFs) appear to be an environmentally friendly option, especially if the coordination metal is a trace element^6,7^ that is easily incorporated by weeds and thus provides an easy way to introduce the potent herbicide contained inside. Apart from bioavailability, the water solubility of MOFs can be modified by surface deposition of polar molecules, which enhance the distribution in soil media.^8,9^ In addition, the porous structure imparts stability by avoiding chemical modification of the encapsulated compounds in the empty voids of the architecture.^10^

The work described here offers a new perspective on the application of MOFs, namely, their implementation in agriculture. In particular, we have developed zinc zeolitic imidazolate frameworks (ZIF-8) with two of the most promising *ortho-*disubstituted disulfides encapsulated within. The resulting structures were characterized by ultrahigh resolution imaging and analytical scanning transmission electron microscopy (STEM) to reveal the spatial arrangement of the herbicide within the MOF host structure. Surface modification employing 2-hydroxypropyl-β-cyclodextrin (HP-β-CD) was carried out to boost the water solubility. The encapsulation percentage and water solubility were subsequently analyzed by a high-performance liquid chromatography (HPLC) method to evaluate the improvements in the properties caused by encapsulation. The novel architectures developed here also showed impressive growth inhibition results against *Lollium rigidum* Gaudin, *Echinochloa crus-galli* (L.) and *Amaranthus Viridis*,^11^ the main weeds that affect southern European crops.

## Results and Discussion

The zinc imidazolate framework (ZIF-8) was synthesized by an *in situ* method in which the disulfide agrochemicals were present in the medium during the MOF formation. The use of Liédana’s method^12^ ensured entrapment of the bioactive compound in the voids present in the icosahedral MOF. **DiS**–**NH**_**2**_ was selected from the disulfide collection due to its simplicity and easy synthesis procedure. Most disulfide compounds are structurally analogous and we therefore employed the information on the encapsulation of this molecule to be applied to the most promising herbicides, such as acetyl derivatives. **DiS**-***O***-**acetyl** was chosen because of its high growth inhibition activity against *L. rigidum* and *E. crus-galli* (the main weeds that infect rice, corn, and potato crops)^11^

**Figure 1:**
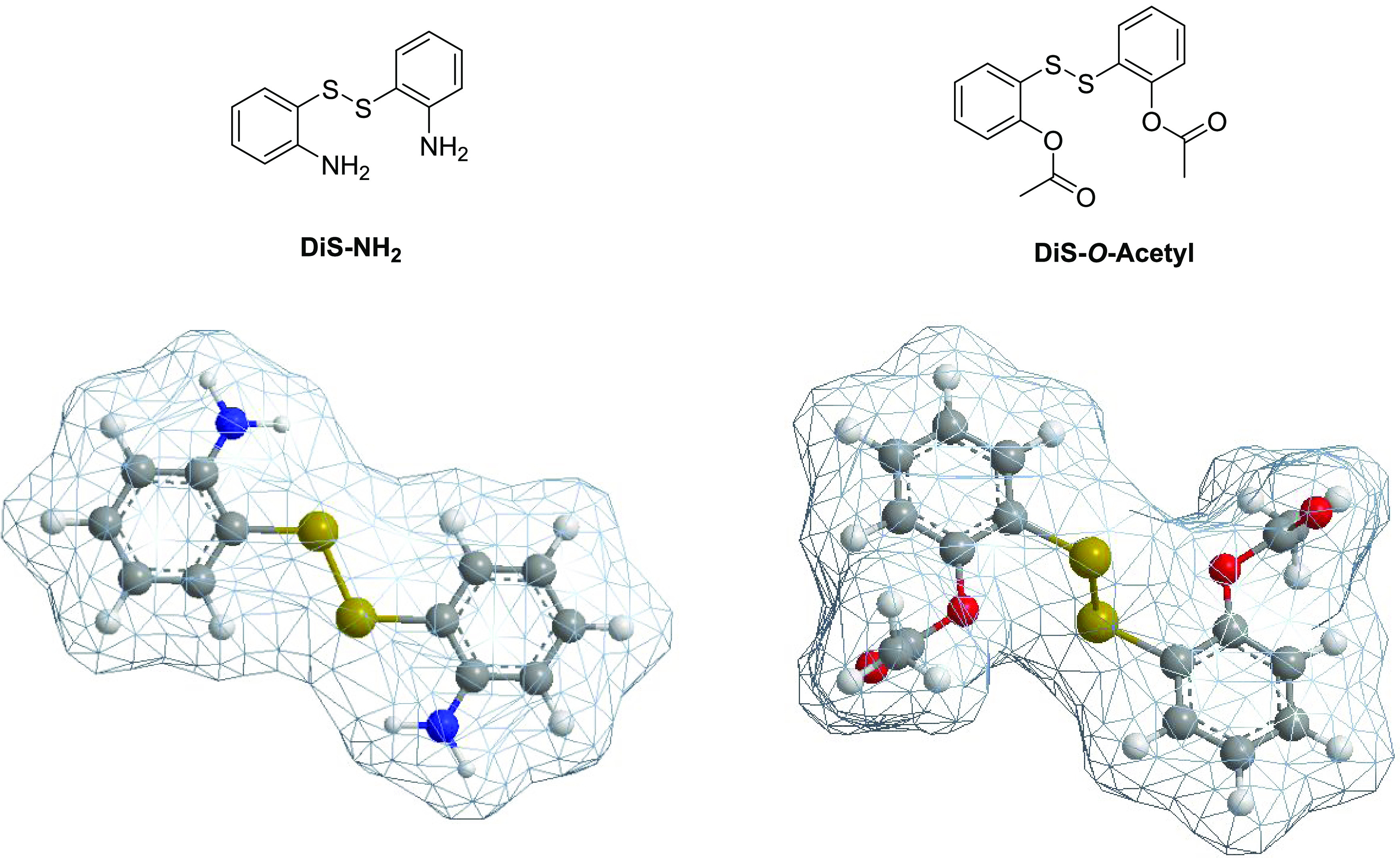
*Ortho-*disubstituted disulfides selected for encapsulation in zinc imidazolate frameworks and their molecular volumes.

X-ray diffraction (XRD) analysis did not show any difference in the crystalline phase on comparing the vacant MOF and the system with **MOF@DiS**–**NH**_**2**_ (Figures S1 and S2). This result shows the high purity of the icosahedral structure of **MOF@DiS**–**NH**_**2**_ and also demonstrates that the *in situ* loading process did not modify the crystal arrangement of the ZIF-8. According to the literature,^13,14^ ZIF-8 has a complex structure of small nominal pores with a radius in the range 0.34–0.42 nm.^15^ According to the molecular volume calculated using B3LYP/6-311G(d,p) for **DiS**–**NH**_**2**_ and **DiS**-***O***-**acetyl**, *i*.*e*., 0.232 and 0.378 nm^3^, respectively, the bioactive compounds fit within the MOF channels (Figure 1). This fact is consistent with the results of aforementioned XRD experiments.

**Figure 2:**
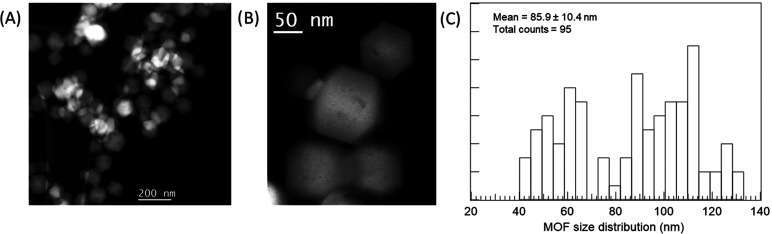
Low (A) and medium (B) magnification HAADF images of the **MOF@DiS**–**NH**_**2**_ sample. (C) MOF particle size distribution histogram.

**Figure 3:**
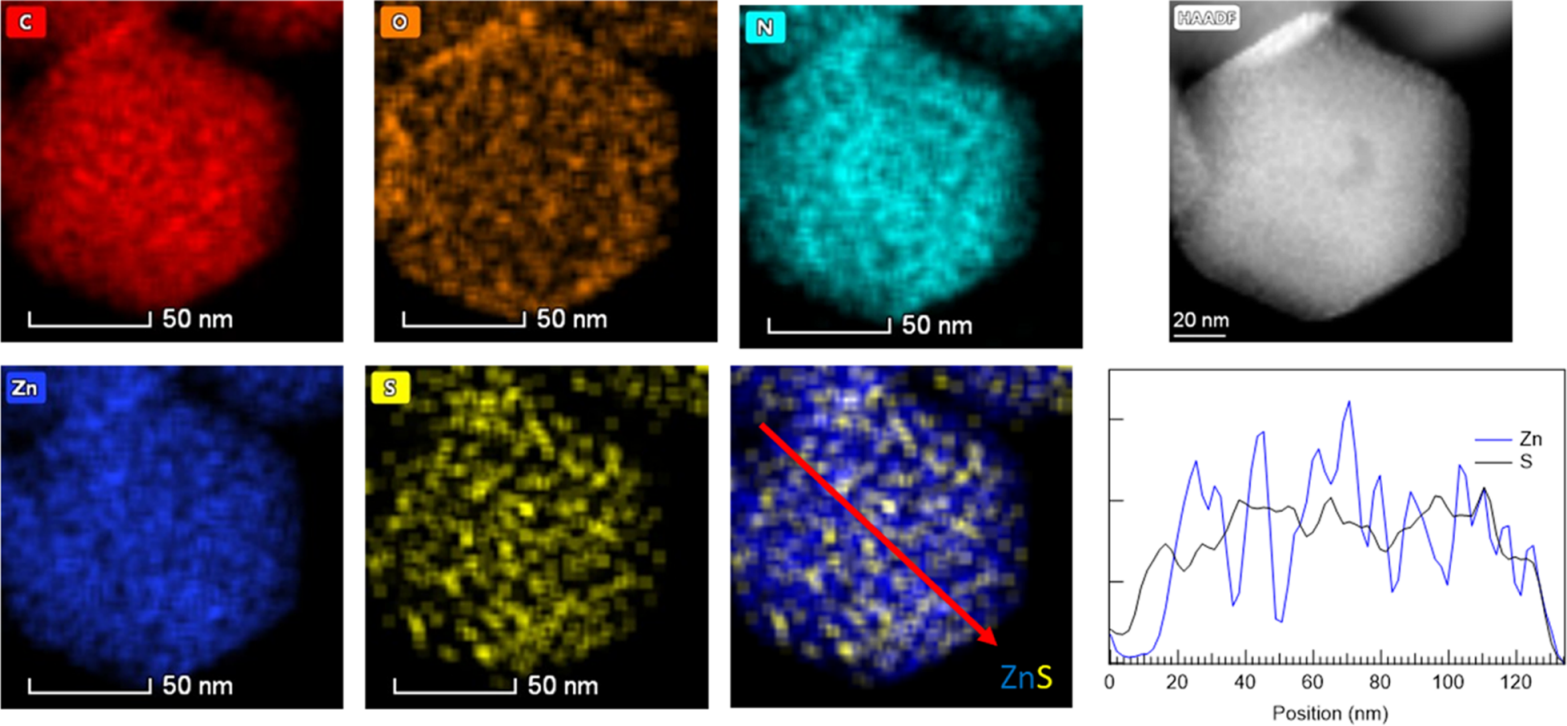
STEM-XEDS chemical maps and HAADF image of MOF@DiS–NH_2_. The Zn and S intensity profiles extracted from the red arrow marked on the Zn–S map (Zn is shown in blue and S in black).

Analysis of **MOF@DiS–NH**_**2**_ by transmission electron microscopy provided structural and chemical information about the sample. In particular, low-magnification high-angle annular dark-field (HAADF) images of the **MOF@DiS–NH**_**2**_ sample are displayed in Figure 2A,B. Note that the MOF crystallite has a rhombic dodecahedral morphology and some heterogeneity in terms of their size. An MOF particle size histogram obtained by measuring 95 nanostructures is represented in Figure 2C. A bimodal distribution with average values of roughly 60 and 113 nm is observed. X-ray energy-dispersive spectrometry (XEDS) analysis of the **MOF@DiS**–**NH**_**2**_ sample (Figures 3 and S3) illustrates the spatial distribution of C (0.277 keV), N (0.392 keV), O (0.523 keV), Zn (8,639 keV), and S (2.307keV). The S and Zn signals are anticorrelated, as evidenced by the S and Zn intensity profile extracted from the area marked in red in the Zn–S combined chemical map. One must bear in mind that Zn is a part of the ZIF-8 MOF structure, whereas S is a part of the encapsulated **DiS**–**NH**_**2**_ molecule.

**Figure 4:**
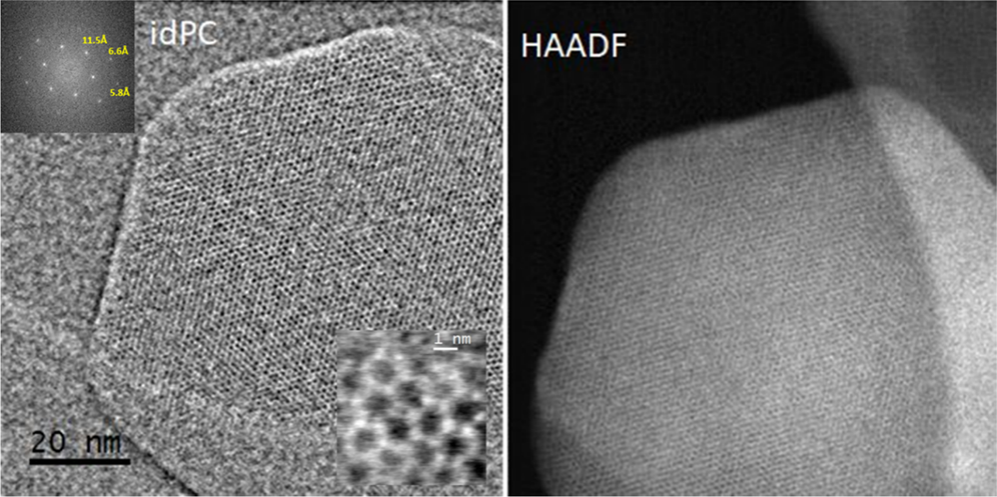
High-resolution high-angle annular dark-field scanning transmission electron microscopy (HR HAADF-STEM) and integrated differential phase contrast (iDPC) images of **MOF@DiS–NH**_**2**_. Enlargement and the digital diffraction pattern (DDP) of the whole image are shown as insets in the iDPC image.

In terms of crystallinity, it has previously been demonstrated that the structures of MOFs are immediately destroyed under high-energy electron beams. In particular, Zhu et al.^16^ reported dose-dependent electron diffraction patterns of ZIF-8 crystals in a study that combined the acquisition of a series of TEM images at a high frame rate (40 fps) with direct-detection electron counting. The results provided evidence that the crystals begin to lose crystallinity when the accumulated dose reaches a value as low as 25 e^–^·Å^–2^ and that they are completely destroyed at a dose of 75 e^–^·Å^–2^. In our study, in order to determine the crystal structure of **MOF@DiS**–**NH**_**2**_, we employed a combination of high-resolution high-angle annular dark-field-scanning transmission electron microscopy (HR HAADF-STEM) and integrated differential phase contrast (iDPC) imaging techniques using a current of around 0.5pA (60 e^–^·Å^–2^) to minimize sample damage. The HR HAADF and the corresponding iDPC images of **MOF@DiS**–**NH**_**2**_ are shown in Figure 4. The observed structure is as one would expect for ZIF-8 with a sodalite topology and a *I*4̅3*m* (#217) space group, with large cages (11.6 Å in diameter) connected through six-membered-ring windows (3.4 Å).

The encapsulation percentages achieved are listed in Table 1. The water solubility was enhanced by 1.4 and 2.5 times for **DiS**–**NH**_**2**_ and **DiS**-***O***-**acetyl**, respectively. The HPLC method for quantification involved dissolving the sample in methanol and applying ultrasonication to destroy the MOF and deliver all of the bioactive molecule contained with the framework.

**Table 1:** Encapsulation Percentages and Water Solubility Enhancement of the Encapsulated Compounds^a^

compounds	encapsulation percentage (%)	solubility enhancement (%)
MOF@DiS–NH_2_	42.80 ± 0.09	140.73 ± 0.03
MOF@DiS-*O*-acetyl	16.71 ± 0.07	252.91 ± 0.72

a Latter are expressed as relative values with respect to those of the free disulfides.

The higher molecular volume of **DiS**-***O***-**acetyl** could explain its lower encapsulation percentage. A small number of molecules are required to fill the MOF pores. With regard to the water solubility enhancement, **DiS**-***O***-**acetyl** showed a more marked improvement and this is due to its lower initial solubility.

**Figure 5:**
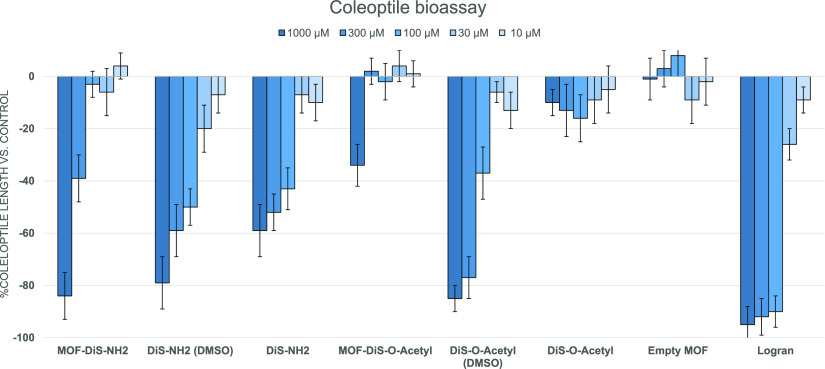
Results of a coleoptile bioassay at pH 7.0 during 24 h.

All of the compounds were tested in a coleoptile bioassay as a first approach to evaluate their phytotoxicity. Nevertheless, the water solubility improvement of the encapsulated compounds was not sufficient to enable dissolution of all of the samples and this led to lower activity than expected. It can be seen from Figure 5 that none of the MOFs boosted the phytotoxicity when the bioassay was carried out at pH 7.0 during 24 h.

**Figure 6:**
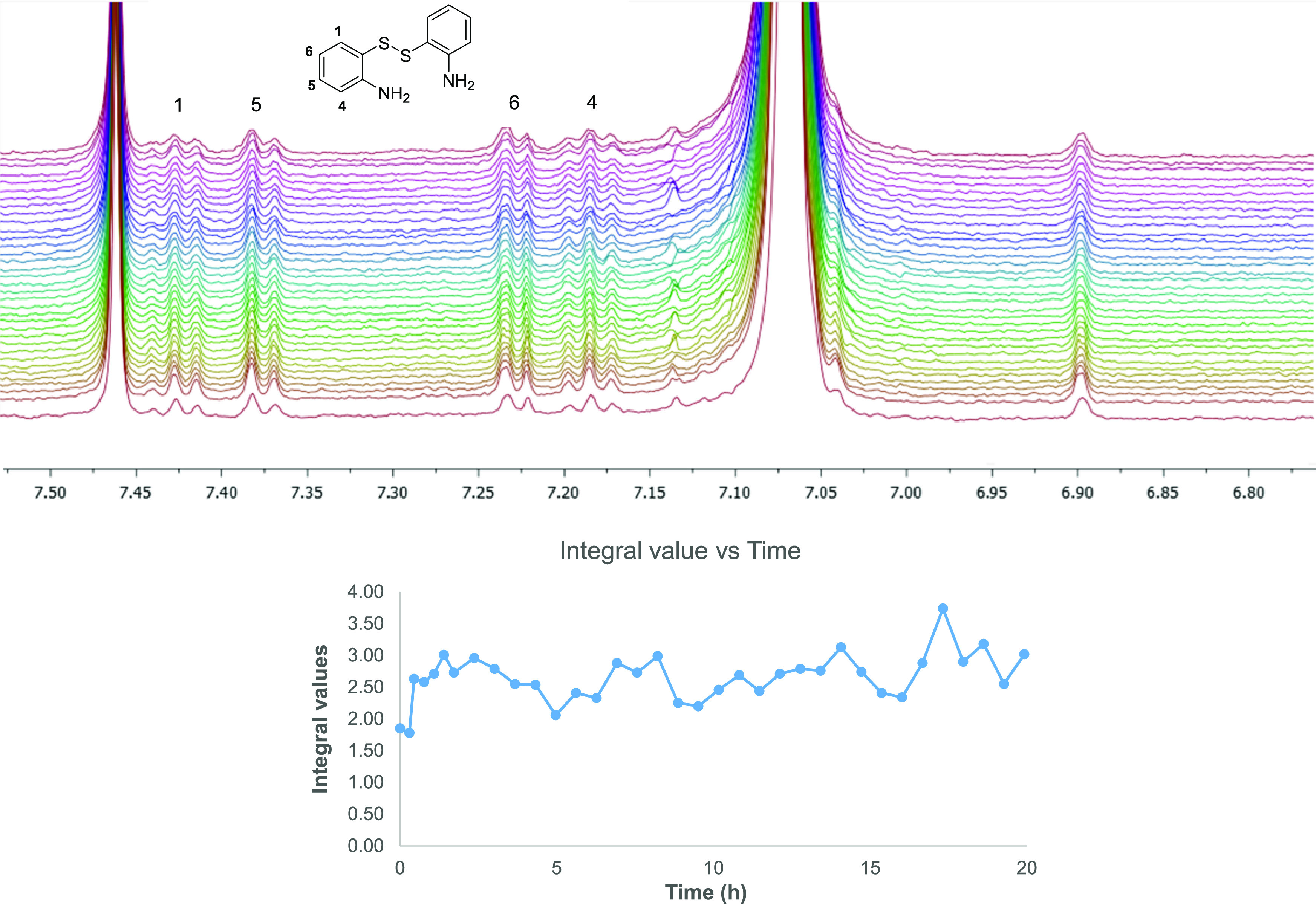
(Top) ^1^H NMR kinetic study to analyze the integral value with time. (Bottom) Changes in the integrals with time.

A ^1^H NMR kinetic study during 24 h at pH 7.0 in distilled water was carried out, with spectra recorded every 15 min during the first hour and then one per hour up to 24 h. The results indicate that the integral values of the signals for H-1, H-4, H-5, and H-6 of **DiS**–**NH**_**2**_ did not change over time when the compound was encapsulated within the MOF (Figures 6 and S4).

This result could be interpreted in two different ways. First, the water solubilities of **MOF@DiS**–**NH**_**2**_ and **MOF@DiS**-*******O***-**acetyl** in the buffer solution used in the bioassay and in the water solution of the NMR probe were quite low. Alternatively, the exposure time and acidity of the media were not sufficiently long or high, respectively, as suggested by other authors.^4^

Given the low water solubility of ZIF-8 structures previously reported in the literature,^17,18^ surface modification appears to be a reasonable approach to solve this solubility problem. This postsynthesis strategy should fulfill three goals. First, it should minimize the tendency of the compounds to aggregate, which would limit the water solubility. In addition, it should control and modulate the delivery rate of the encapsulated compounds but it should not block the pores that contain the disulfides. Finally, it should improve the biorecognition and targeting abilities with respect to the unmodified architecture. According to these requirements, 2-hydroxypropyl-β-cyclodextrin (HP-β-CD) appeared to be a perfect candidate for use as a functionalizing agent.

**Figure 7:**
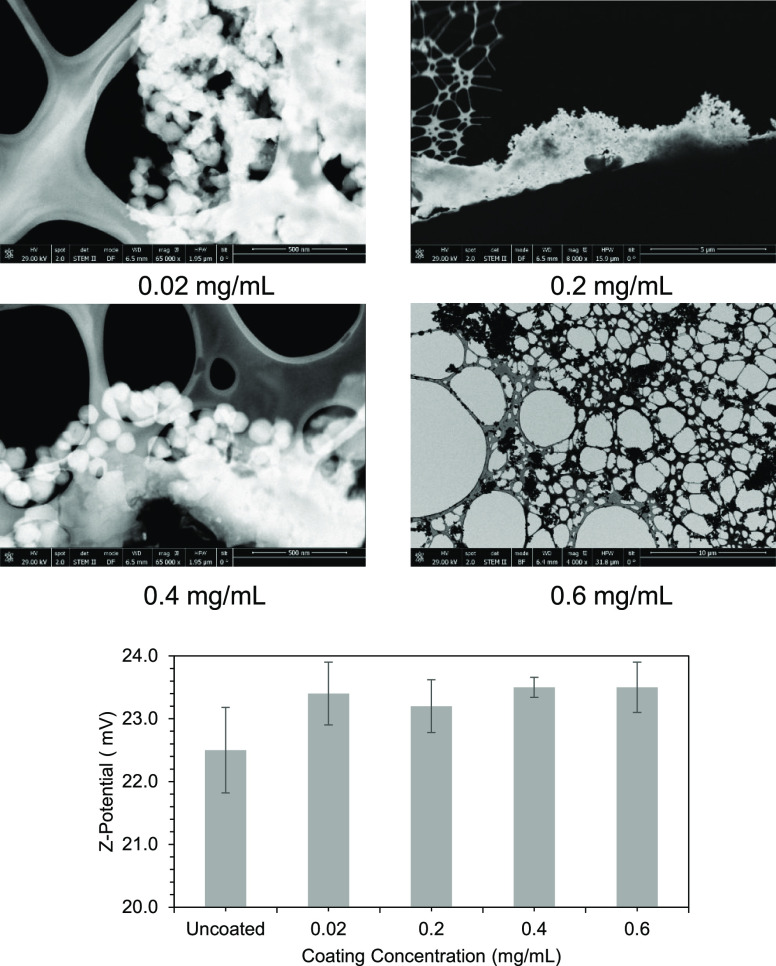
(Top) SEM images of the samples modified using different concentrations of the surface functionalizing agent. (Bottom) ζ-potential values for each concentration.

The two encapsulated (**MOF@DiS**–**NH**_**2**_ and **MOF@DiS**-***O***-**acetyl**) compounds were submitted to the functionalization process. Different concentrations of HP-β-CD were tested (0.6, 0.4, 0.2, and 0.02 mg/mL) in order to identify the best conditions to obtain the expected properties. In particular, the ζ-potential was measured by electrophoretic light scattering to determine the appropriate concentration to avoid the bridging effect toward aggregation.^8^ In contrast to the situation observed for the uncoated loaded MOFs, all of the cyclodextrin concentrations showed positive values in the range of 23–24 mV (Figure 7), which according to the bilayer model involves a negatively charged second layer; so, positive particles would be attracted by the MOF surface. In spite of the small differences between the concentrations of HP-β-CD, the SEM images in Figure 7 confirm that only at 0.6 mg/mL, the MOF crystallites became well dispersed.

It has previously been suggested that HP-β-CD charges the surface of the MOFs regardless of its concentration, but at low concentration, the cyclodextrin attempts to remain in contact with the maximum number of MOF crystallites. As a result, only with high concentrations of the coating molecule the bridging effect can be effectively avoided. Comparison of the results of water solubility tests of the uncoated samples and those coated with 0.6 mg/mL HP-β-CD (see Table 2) indicates an impressive increase in the solubility after the surface modification step. Both samples were fully dissolved in water and the improvements, with respect to the uncoated samples, were by six and four times, respectively.

**Table 2:** Comparison of the Water Solubility Improvement in the Uncoated and 0.6 mg/mL HP-β-CD-Coated Samples

compound	solubility enhancement (coated) (%)	solubility enhancement (uncoated) (%)
MOF@DiS–NH_2_	833.63 ± 9.72	140.73 ± 0.03
MOF@DiS-*O*-acetyl	997.68 ± 12.44	252.91 ± 0.72

**Figure 8:**
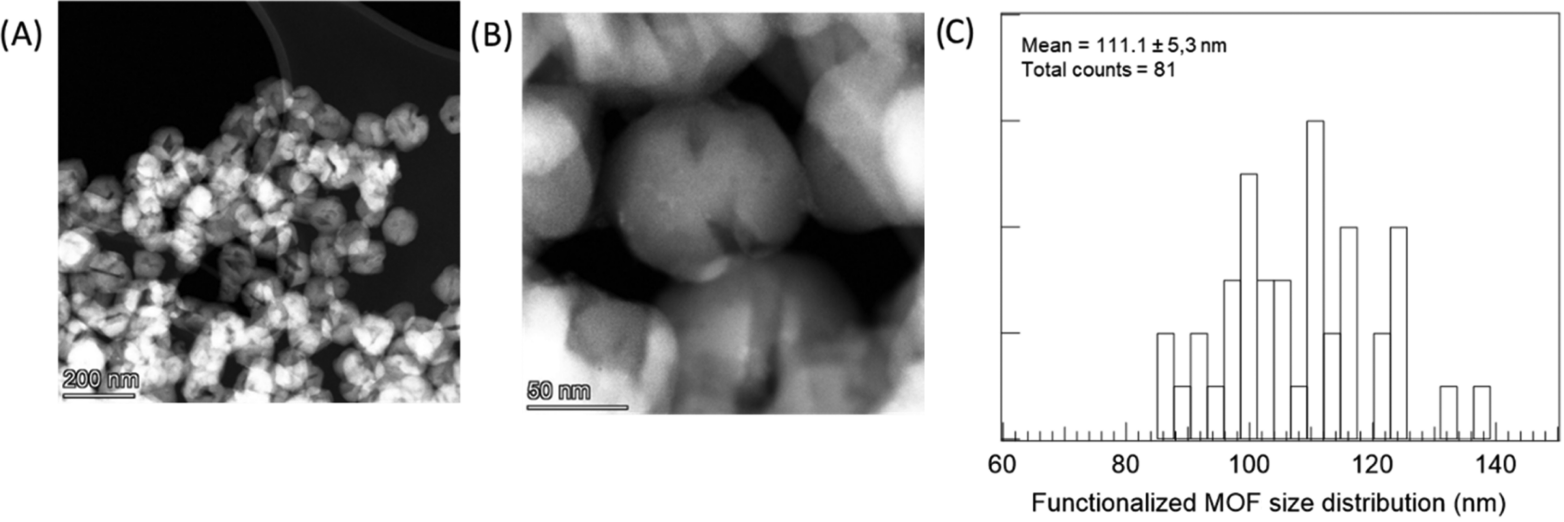
Low (A) and medium (B) magnification HAADF images of **MOF@DiS**–**NH**_**2**_ functionalized with 0.6 mg/mL HP-β-CD. (C) Crystallite size histogram of the **functionalizedMOF@DiS**–**NH**_**2**_ sample.

**Figure 9:**
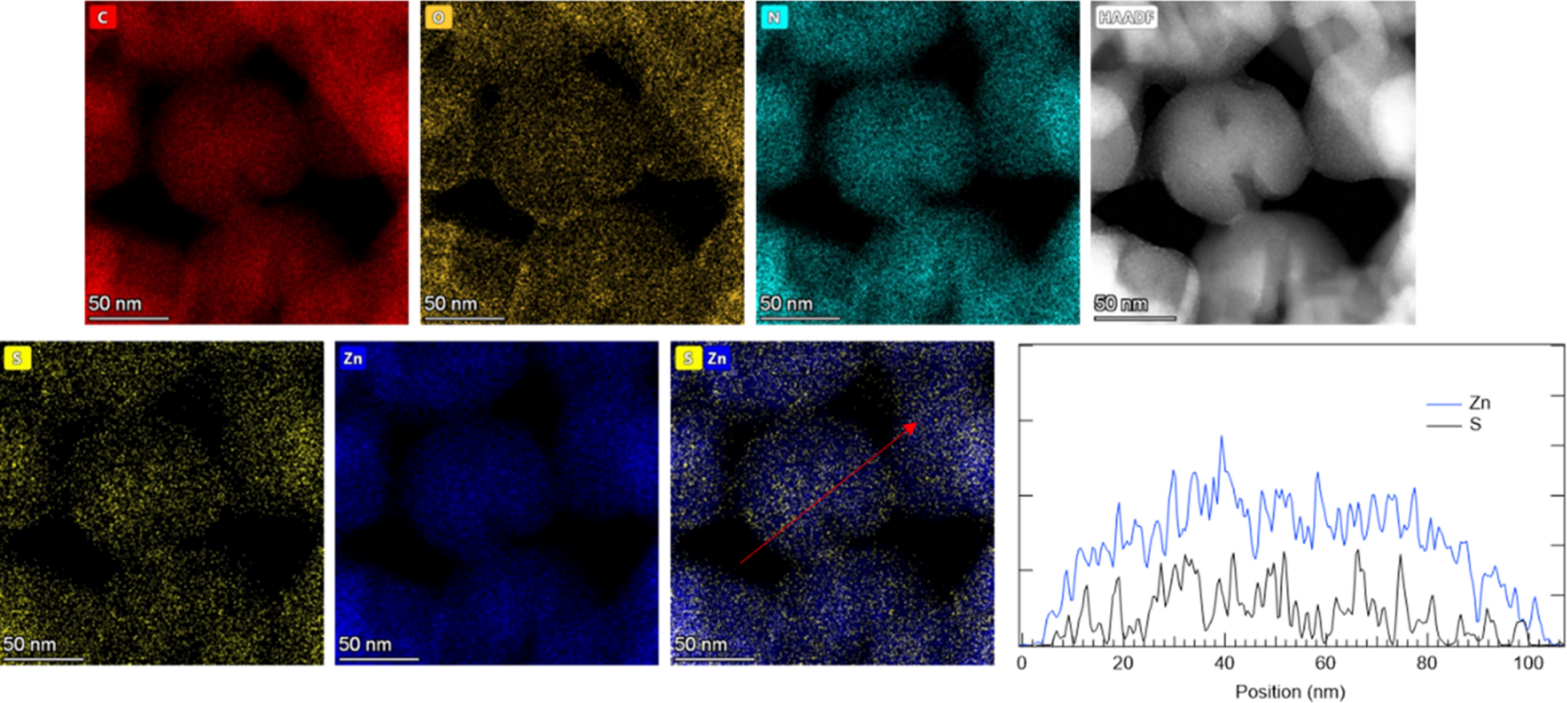
STEM-XEDS chemical maps and HAADF image of the **functionalizedMOF@DiS**–**NH**_**2**_ sample. Zn and S intensity profiles along the red arrow marked on the combined Zn–S map are also displayed.

Scanning transmission electron microscopy analysis of the **MOF@DiS**–**NH**_**2**_ sample modified with 0.6 mg/mL HP-β-CD (functionalized **MOF@DiS**–**NH**_**2**_) indicates a variation in the MOF crystallite morphology. Low and medium magnification HAADF images are shown in Figure 8 along with the corresponding particle size histogram after measuring 81 nanostructures, which could be fitted to a normal distribution with a mean value of 111 nm. Comparison with **MOF@DiS**–**NH**_**2**_ (nonfunctionalized) indicates an increase in the MOF particle size, which is an expected change due to the functionalization process. In terms of chemical composition, EDX chemical maps (Figures 9 and S3) show results similar to those obtained for the nonfunctionalized samples. In this case, an anticorrelation can again be observed between S and Zn signals.

**Figure 10:**
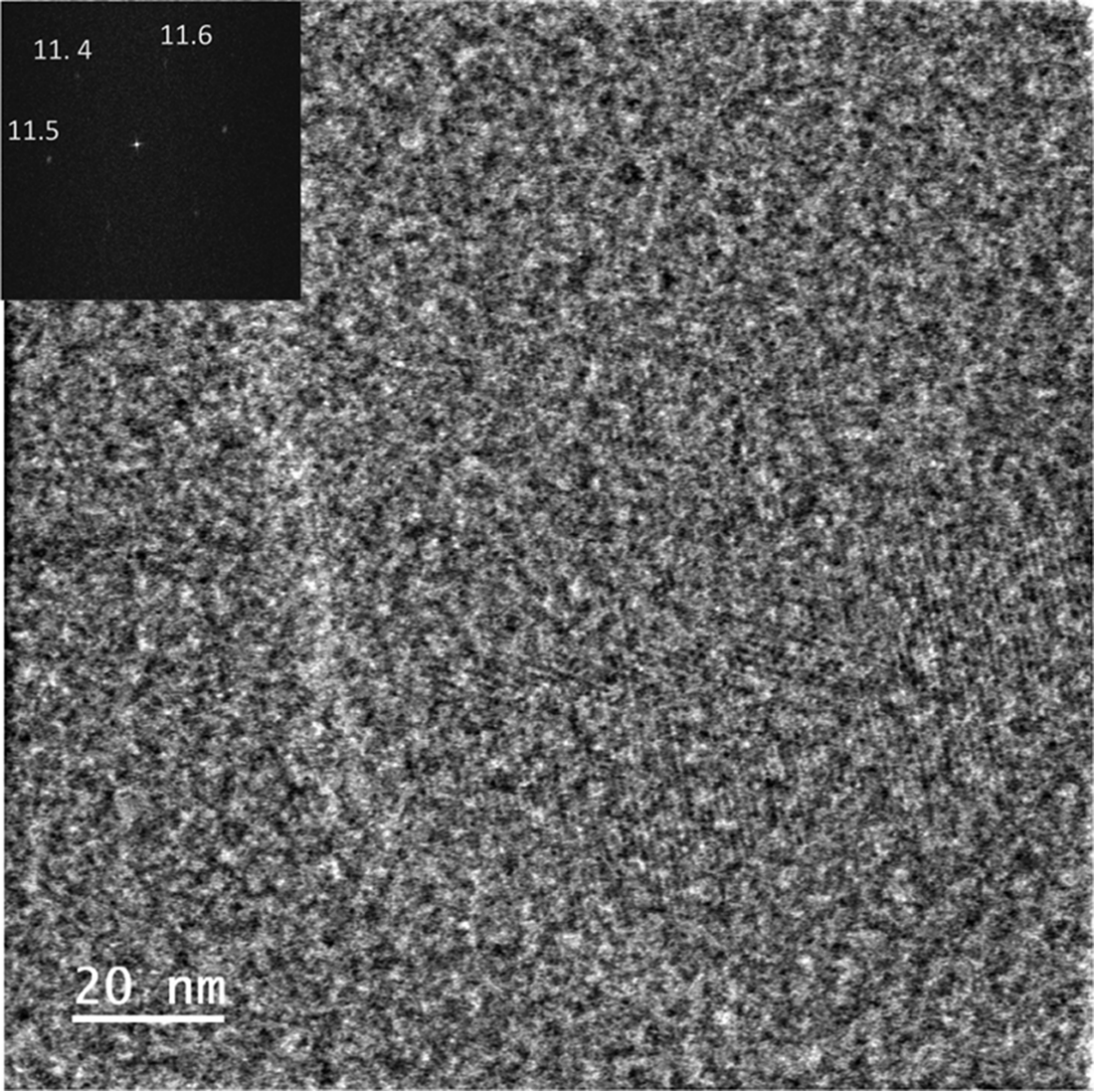
Integrated differential phase contrast (iDPC) image recorded on the **functionalizedMOF@DiS**–**NH**_**2**_ sample. The DDP of the whole image is shown as an inset.

The presence of a thick functionalizing shell surrounding the crystallites of the *ortho*-disulfide-loaded MOFs adds an amorphous-like background to the integrated differential phase contrast (iDPC) images, which makes the appreciation of the crystalline structure of the sample difficult with the naked eye. Note that, although the high-resolution detail is still observable in the experimental iDPC images of the coated samples (Figure 10), such detail can be much more clearly observed in the digital diffraction patterns (DDPs) of the images. Moreover, the quantitative analysis of the reflections observed in these patterns reveals that the surface functionalization did not induce any structural change. Thus, the DDP shown as an inset in Figure 10 depicts the hexagonal arrangement of reflections at roughly 11.5 Å, which is characteristic of the [111] zone axis of the ZIF-8 structure and is similar to that observed in the case of the uncoated sample (Figure 4).

**Figure 11:**
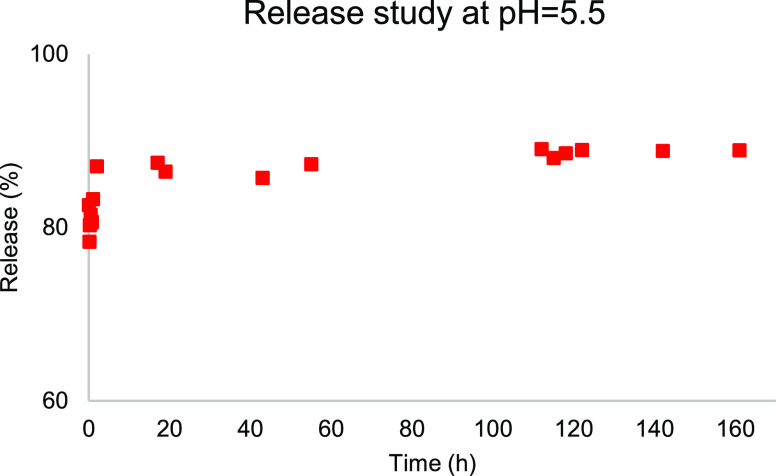
HPLC release study of **DiS**–**NH**_**2**_ from 0.6 mg/mL HP-β-CD **functionalizedMOF@DiS**–**NH**_**2**_.

The ability of the **MOF@DiS**–**NH**_**2**_ sample coated using 0.6 mg/mL HP-β-CD to deliver the bioactive molecule was determined by an HPLC release study in distilled water at pH 5.5 (Figure 11). The experiment was carried out for 7 days. In this case, the fully solubilized sample showed a fast release during the first 2 h at pH 5.5, in clear contrast with the kinetic study carried out on the uncoated sample. The bioactive encapsulated disulfide samples seem to be stable during at least 7 days. After 9 months of storage, the samples degraded, a result that indicates the biodegradability of these systems—a prerequisite for a safe herbicide (Figure S5).

**Figure 12:**
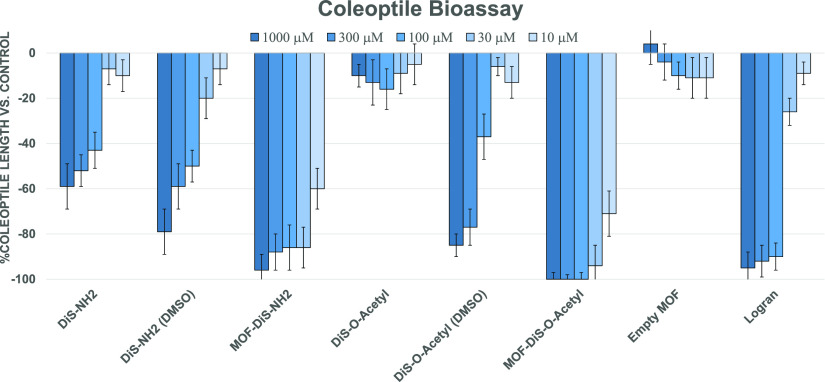
Coleoptile bioassay at pH 5.6 during 48 h.

Once the architecture of the final hybrid metal–organic materials had been achieved, wheat coleoptile bioassays were carried out to evaluate the phytotoxicity in a first approach. In this case, more acidic conditions at pH 5.6 (still within the limits of wheat viability) and 48 h were employed while ensuring that the control fulfilled the general specifications.^19^ The phytotoxicity of the MOFs exceeded even that observed on using an organic solvent to dissolve the samples (Figure 12). In the case of **MOF@DiS**-***O***-**acetyl**, growth inhibition of over 95% was observed at a concentration of only 30 μM. Both MOF samples displayed more than 60% inhibition at concentrations in the nanomolar range (100 nM). In terms of IC50 values, which represent the necessary concentration to achieve 50% inhibition, values for **MOF@DiS**–**NH**_**2**_ (IC_50_ = 5.413 μM, *R*^2^ = 0.9856) and **MOF@DiS**-***O***-**acetyl** (IC_50_ = 3.892 μM, *R*^2^ = 0.9964) were substantially higher than that of the commercial herbicide Logran (IC_50_ = 43.46 μM, *R*^2^ = 0.9948).

These results confirm that functionalization provided all of the expected properties described above. As previously suggested,^20^ the small size of the MOF crystallites and the recognition at the cellular wall of the vegetable cell of the glucose units in the cyclodextrins allow them to cross toward the cytosol, where the encapsulated biomolecules are delivered. Solubilization and biorecognition of the coating agent have therefore boosted all of the appropriate physicochemical properties of the MOF and free disulfide samples.

More specific bioassays were carried out on the most relevant weed species that infect the main worldwide crops: rice, corn, and potatoes. Both **MOF@DiS**–**NH**_**2**_ and **MOF@DiS**-***O***-**acetyl** can be considered as stable, nonpersistent, and water-soluble candidates for the effective fight against these pests.

**Figure 13:**
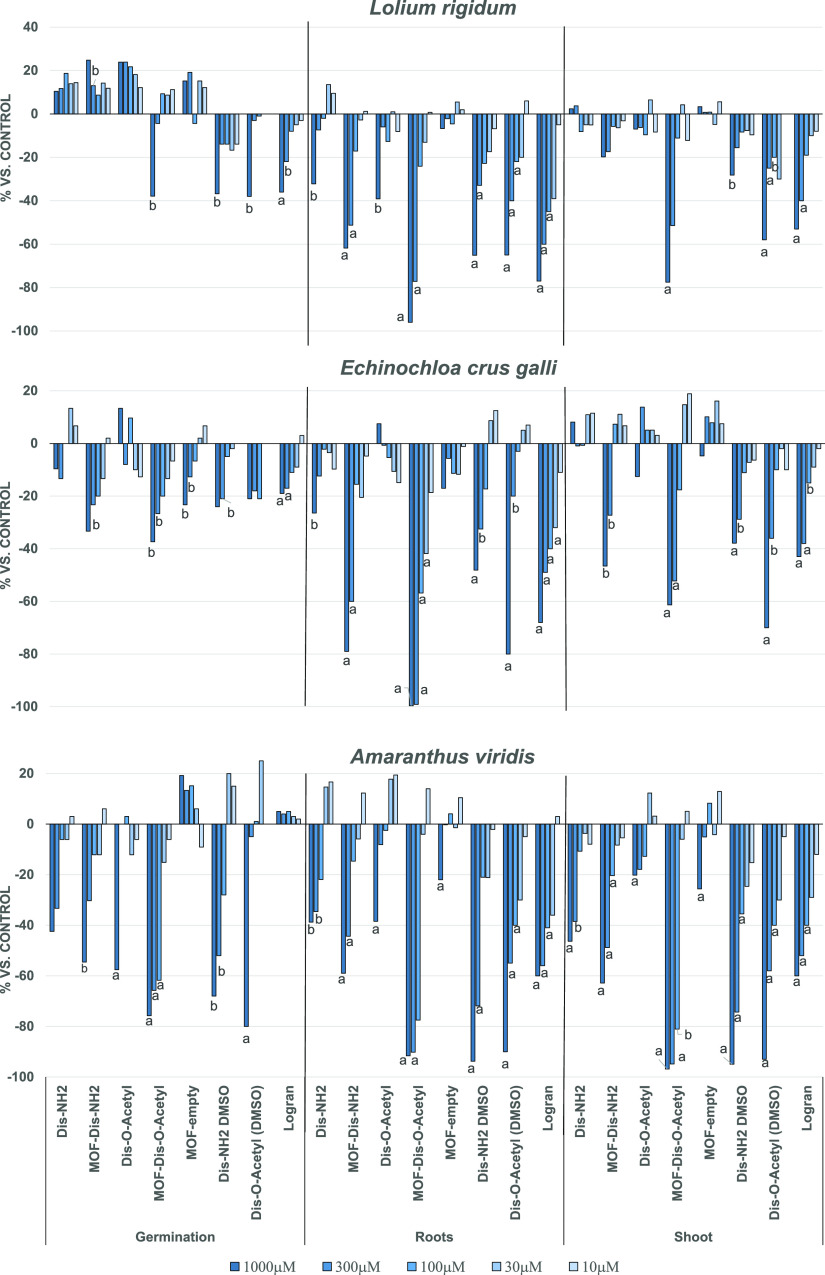
Results of the phytotoxicity bioassay against *E. crus-galli*, *A. viridis*, and *L. rigidum*. Positive values indicate stimulation of growth vs the control and negative values indicate inhibition. Significance levels *p* < 0.01 (a) or 0.01 < *p* < 0.05 (b).

The results related to the influence of the encapsulated compounds in functionalized metal–organic frameworks on germination, root growth, and stem development in weed species are shown in Figure 13. In these tests, the samples were dispersed in water, except in the case of samples with “dimethyl sulfoxide (DMSO)” in their name, which were predissolved in the organic medium. The philosophy behind the bioassay was to achieve a strong negative growth percentage by employing water as the solvent since the use of organic media (DMSO) is clearly not a green approach in the particular case of agricultural uses. Functionalization after encapsulation and the use of organic compounds and oligo elements such as zinc seem to be key, as evidenced by the graphs above.

The results gathered in Figure 13 show that the bioactive agents **DiS**–**NH**_**2**_ and **DiS**-***O***-**acetyl** have more potent effects as herbicides when they are encapsulated in the functionalized MOFs when compared to solutions in an organic medium (DMSO). Furthermore, the activity values corresponding to the “empty MOF” confirm that the capsule is innocuous to these plants. Importantly, it is worth noting that the results for Logran, which is a positive control employed to analyze the performance of the bioassay, were surpassed by the functionalized **MOF@DiS**–**NH**_2_ and **MOF@DiS**-***O***-**acetyl** samples. All of the weeds seem to be mainly affected in their root production, with **DiS**-***O***-**acetyl** showing a specific preference for this part of the plant regardless of the weed. Despite the fact that the two disulfides express their bioactivity after encapsulation, the case of the acetyl derivative is more pronounced. This finding indicates that the solubility enhancement (see Table 2) is clearly a physicochemical property that is directly related to bioactivity. Thus, root inhibition by functionalized **MOF@DiS**-***O***-**acetyl** exceeds 80% in all cases at the 300 μM level, which, in fact, corresponds to a very low concentration.

It also appears clear that *A. viridis* is the most susceptible weed to the new herbicide synthesized, even in the germination parameter analysis, which is usually the parameter that fluctuates the most. In addition, *E. crus-galli* and *L. rigidum* show the same profile as *Amaranthus* in the case of roots and stem, where MOF encapsulation leads to higher activity than the compounds dissolved in DMSO. These results indicate that the enhancement of the water solubility is not the only crucial factor but also the transport action of metal–organic frameworks. Recognition of glucoside fragments on the MOF surface seems to facilitate assimilation by vegetable cells. The protection offered by the encapsulating agent also prevents the occurrence of side reactions on the disulfides, thus ensuring correct transport from the solution to the active site. Moreover, the biocompatibility of ZIF-8 toward cell conditions, as reported previously in the literature, plays an important role.^17,21^

## Conclusions

Natural herbicide models have been successfully encapsulated at 25 °C in a single-step process. The solubility and bioactivity were enhanced in the fight against the main weeds that infect rice, potatoes, and corn. Functionalization of the MOF is the key to make bioherbicide available to farmers as an alternative to traditional herbicides based on halogenated compounds or heavy metals. In the case of disulfide derivatives, which have proven potent phytotoxic activity, we achieved root growth inhibition over 80% for all of the weeds tested. High-resolution scanning transmission electron microscopy studies revealed the incorporation of the **DiS**–**NH**_**2**_ molecules inside the pores of MOF crystallites, which have a crystal structure based on a sodalite topology. Furthermore, functionalization of the samples with HP-β-CD did not modify the crystal structure but enhanced the water solubility by almost 10 times. The transport properties of the ZIF-8 structure also boosted the inhibitory activity of the **DiS**–**NH**_**2**_ and **DiS**-***O***-**acetyl** against the main weeds of rice, potatoes, and corn, with higher value—in some cases double—results obtained with a commercial herbicide.

## Supporting Information Available

Detailed experimental procedures; XRD, EDS, NMR, and SEM spectra data and images are included; and all HPLC methods and results (PDF)
