# Supplementary material for: One-Step Encapsulation of ortho-Disulfides in Functionalized Zinc MOF. Enabling Metal–Organic Frameworks in Agriculture
Source: ACS Appl Mater Interfaces. 2021 Feb 12;13(7):7997–8005. doi: 10.1021/acsami.0c21488 (PMC8637546; doi:10.1021/acsami.0c21488)
Supplement: Supplementary file 1 — am0c21488_si_001.pdf [file am0c21488_si_001.pdf]

Supporting Information

One-Step Encapsulation of *Ortho*-Disulfides in Functionalized Zinc MOF. Enabling Metal-Organic Frameworks in Agriculture.

Francisco J. R. Mejías[a], Susana Trasobares[b], Rosa M. Varela[a], José M. G. Molinillo[a], José J. Calvino[b], Francisco A. Macías[a].*

[a]Allelopathy Group, Department of Organic Chemistry, Institute of Biomolecules (INBIO), Campus CEIA3, School of Science, University of Cádiz, C/ República Saharaui, 7, 11510, Puerto Real (Cádiz), Spain.

[b]Departamento de Ciencia de los Materiales e Ingeniería Metalúrgica y Química Inorgánica, Facultad de Ciencias, Universidad de Cádiz, C/ República Saharaui, 7, 11510, Puerto Real (Cádiz), Spain

* Email: famacias@uca.es

Table of Contents

Experimental Procedures **S-3**

Figure S1. X-Ray diffraction spectra **S-8**

Figure S2. X-Ray diffraction spectra **S-8**

Figure S3. EDS spectra **S-9**

Figure S4. ^{1}H-NMR spectra **S-10**

Figure S5. SEM images of MOFs **S-11**

Table S1. HPLC method data **S-11**

Table S2. HPLC method data **S-12**

Table S3. HPLC results **S-13**

Table S4. HPLC results **S-14**

Table S5. HPLC results **S-14**

Table S6. HPLC results **S-15**

Experimental procedures

Synthesis of 2,2'-disulfanediyldianiline (DiS-NH_2) and disulfanediylbis(2,1-phenylene) diacetate (DiS-*O*-Acetyl). The method described by Mejías *et al.* was employed[4].

Synthesis of MOF@ DiS-NH_2 and MOF@DiS-*O*-Acetyl. The method is a modification of that reported by Liédana[13]. **DiS-NH_2** (0.4 mmoles) was dissolved in MeOH (30 mL) together with $Zn(NO_3)_2$ (1 mmol). This mixture was added dropwise to a solution of 2-methylimidazole (10 mM) in MeOH. The mixture was stirred at room temperature for 2 h. The resulting solution was centrifuged at 15000 rpm for 15 min and the supernatant was removed. The solid precipitate was washed three times with distilled water and dried in a vacuum oven (35 ºC) overnight. Samples were stored at –20 ºC under a nitrogen atmosphere.

Electron microscopy studies. Samples for scanning electron microscopy were prepared by depositing a tiny portion of the solid directly onto a 3 mm, lacey-carbon coated 200 mesh copper grid.

The large area views of the samples were recorded using the scanning transmission electron microscopy (STEM) detector installed on an FEI Nova NanoSEM 450 scanning electron microscope. Bright-field (BF), dark-field (DF), and high-angle annular dark-field (HAADF) images were recorded using the annular-type detector installed on this microscope.

Ultra-high resolution scanning transmission electron microscopy (STEM) studies were performed on a double Aberration-Corrected (AC) FEI Titan Cubed Themis 60-300 microscope operated at 300 kV. The equipment was also equipped with a Super X-G2 X-ray high sensitivity energy-dispersive spectrometer, thus providing a tool to simultaneously combine spectroscopy and image signals.

XEDS analysis was performed by acquiring collections of 80 × 80 pixel XEDS maps of the C (0.277 keV), N (0.392 keV), O (0.523 keV), Zn (8.639 keV) and S (2.307 keV) signals. To improve visualization, the element maps were postfiltered using Average 3, as provided in Velox software.

Due to the high sensitivity of the MOF structures under the electron beam, atomically resolved images were acquired using iDPC imaging. By using the HAADF and FEI DF4 (four segments) detectors, 2048 × 2048 HAADF and iDPC images were recorded simultaneously using a convergence angle of 18.6 mrad and a camera length of 285 mm to optimize the collection of both types of signal. To limit the damage by the electron beam, a fast image recording protocol was used and this combined a beam current of 0.5 pA and a 2.5 μs dwell time.

NMR studies. A ^{1}H-NMR release study was carried out at 25 ºC using D_2O as solvent with an Agilent INOVA spectrometer at 499.719 MHz. Spectra of **MOF@DiS-NH$_2$** were collected every 30 mins. The residual peak for water was referenced to δ 4.79 ppm.

Functionalization of MOF@ DiS-NH$_2$ and MOF@DiS-*O*-Acetyl with 2-hydroxypropyl-β-cyclodextrin. Samples were dispersed in 2 mL of different solutions of HP-β-CD (0.6, 0.2, 0.4 and 0.02 mg/mL). The new solution was shaken during 24 hours and they were then centrifuged at 10000 rpm during 15 minutes. The supernatant was removed and samples were dried overnight in a vacuum oven (35 ºC). Samples were stored at –20 ºC in a nitrogen atmosphere.

ζ-Potential measurements. The stabilities of the encapsulated systems were assessed by measuring the Zeta potential. A Malvern Instruments Zsizer nano system and disposable

folded capillary cells DTS 1070 were employed. Functionalized MOFs with different concentrations of HP-β-CD (0.6, 0.2, 0.4 and 0.02 mg/mL) were analyzed for each sample at 25 °C and pH 7.0. Each value is an average of 100 measurements.

HPLC quantification method and analysis. Calibration curves for **DiS-NH2** and **DiS-*O*-Acetyl** were obtained in MeOH at eight concentrations (300, 200, 100, 50, 25,10, 5 and 1 ppm). VWR Hitachi HPLC equipment was employed, with a diode array as detector (254 nm) and Phenomenex® column with a 5 μm pore size. Goodness of fit values for **DiS-NH2** and **DiS-*O*-Acetyl** were 0.9985 and 0.9988, respectively. The gradient method is shown in Table S1 for **DiS-NH2** and Table S2 for **DiS-*O*-Acetyl**. The retention time, area, mean area and standard deviation for each compound are shown in Table S3 and Table S4.

Water solubility measurements. To analyze the solubility enhancement, **MOF@ DiS-NH$_2$** and **MOF@DiS-*O*-Acetyl** were dispersed in distilled water and shaken with a vortex. After 24 h, the samples were analyzed by HPLC applying the quantification method described previously in order to ascertain the levels of disulfides that could be solubilized. The percentages shown in Tables 1 and 2 (Results and discussion) are the results obtained after applying Equation 1. All of the information related to the the HPLC analysis for the water solubility of the free compounds is provided in Table S5.

$$\text{Solubility enhancement (\%)} = \frac{\text{Water solubility when encapsulated}}{\text{Water solubility of free compound}} \cdot 100 \qquad \text{(Eq. 1)}$$

Encapsulation efficiency. The encapsulation percentage was evaluated by the standard method reported in literature[4]. Organic solvents and ultrasound were applied to break the MOF structure and release all the encapsulated compound. The calculations were carried out using Equation 2. The information on the encapsulation percentage is provided in Table S6.

$$\text{Encapsulation efficiency (\%)} = \frac{\text{[Bioactive compound] encapsulated}}{\text{Total [Bioactive compound] employed in synthesis}} \cdot 100 \text{ (Eq. 2)}$$

Etiolated Wheat Coleoptile Bioassay. The experiments were carried out following the procedure reported in the literature[23] with some modifications. **MOF@DiS-NH$_2$**, **MOF@DiS-*O*-Acetyl**, **DiS-NH2**, **DiS-*O*-Acetyl** and Empty-MOF were added without predissolution in dimethyl sulfoxide (DMSO), but diluted in an aqueous phosphate-buffered saline solution (PBS) containing 2% sucrose at pH 7.0 or pH 5.6 and the corresponding concentrations of the bioactive compound (10, 30, 100, 300 and 1000 μM). The concentrations of the bioactive compound encapsulated within the MOFs were recalculated by applying the encapsulation percentage obtained for every *ortho*-disubstituted disulfide compound. A 10 mM buffer solution was used to avoid osmotic stress.

Phytotoxicity Bioassay. The selection of target plants was based on an optimization process carried out in our search for weed species[24] with some modifications. The monocotyledons barnyardgrass (E. crus-galli L.) and annual ryegrass (Lolium rigidum), and the dicotyledon redroot pigweed (Amaranthus retroflexus L.) were selected. Bioassays were conducted using Petri dishes (50 mm diameter) with one sheet of Whatman No. 1 filter paper as a support. Germination and growth were conducted in aqueous solutions at controlled pH by using 10^{-2} M 2-[N-morpholino]ethanesulfonic acid (MES) and 1 M NaOH (pH 6.0). The compounds to be assayed were dissolved in water only and these solutions were diluted with buffer so that test concentrations for each compound ($10^{-3.3} \times 10^{-4}$, $10^{-4.3} \times 10^{-5}$ and 10^{-5} M) were achieved. Four replicates were used for each weed species, each containing 20 seeds. Treatment, control or internal reference solution (1 mL) was added to each Petri dish. After adding the seeds and aqueous solutions, Petri dishes were sealed with Parafilm® to ensure closed-system models. Seeds were further incubated at 25 °C in a Memmert ICE 700

controlled environment growth chamber. The photoperiod was 16/8 h light/dark for barnyardgrass, annual ryegrass and redroot pigweed. Bioassays took 8 days. After growth, plants were frozen at −10 °C for 24 h to avoid subsequent growth during the measurement process. Evaluated parameters (germination rate, root length, and shoot length) were recorded using a Fitomed system[25], which allowed automatic data acquisition and statistical analysis using its associated software. Data were analyzed statistically using Welch's test, with significance fixed at 0.01 and 0.05. Results are presented as percentage differences from the control. Zero represents control, positive values represent stimulation, and negative values represent inhibition.

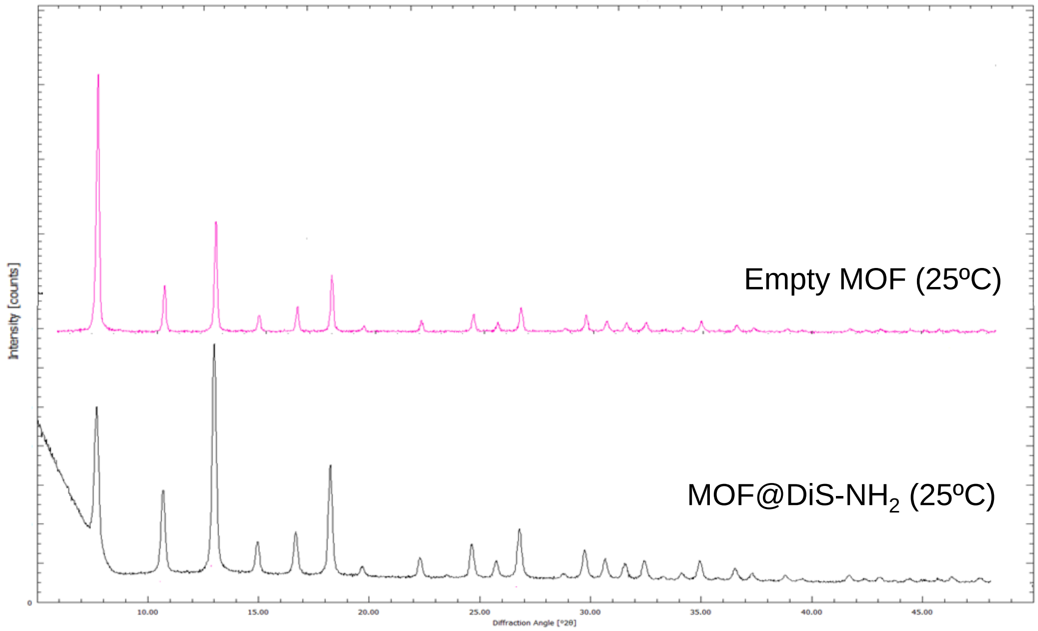

Figure S1. XRD comparison between empty MOF and **MOF@DiS-NH$_2$** at the same temperature.

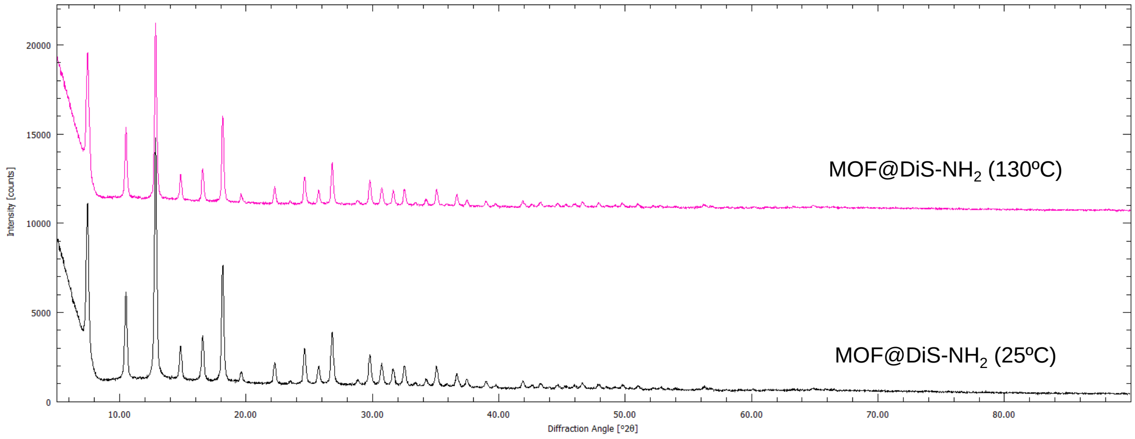

Figure S2. XRD comparison between synthesis temperature of **MOF@DiS-NH$_2$** at 25ºC and 130ºC.

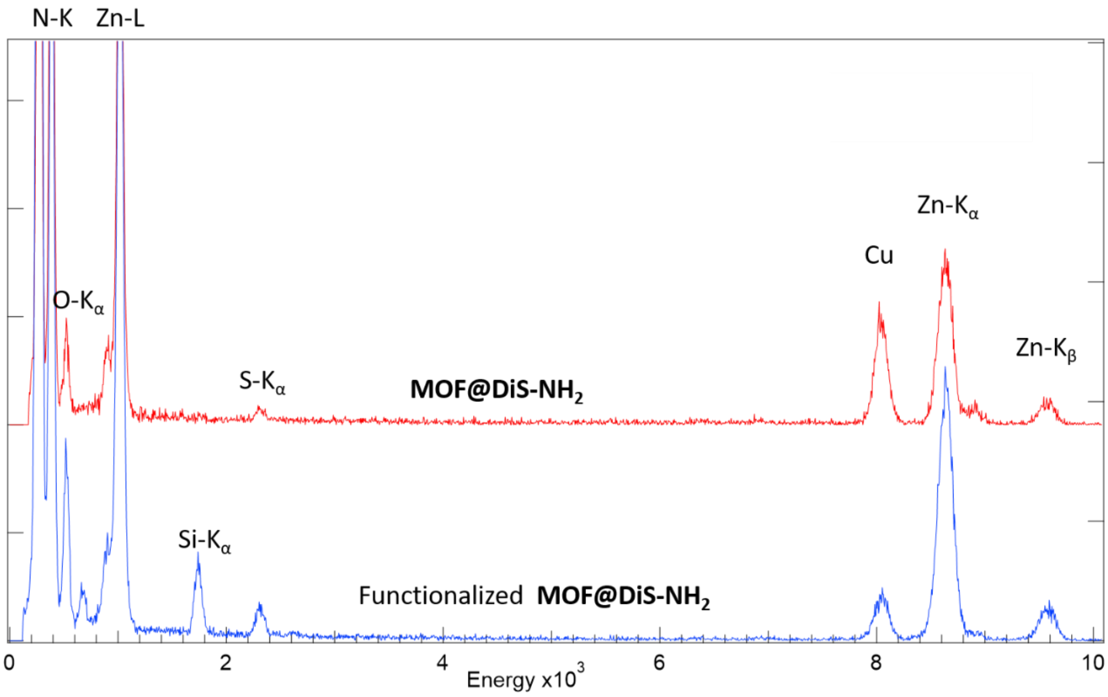

Figure S3. EDS spectra corresponding to the MOF@DiS-NH_2 and functionalized MOF@DiS-NH_2 samples.

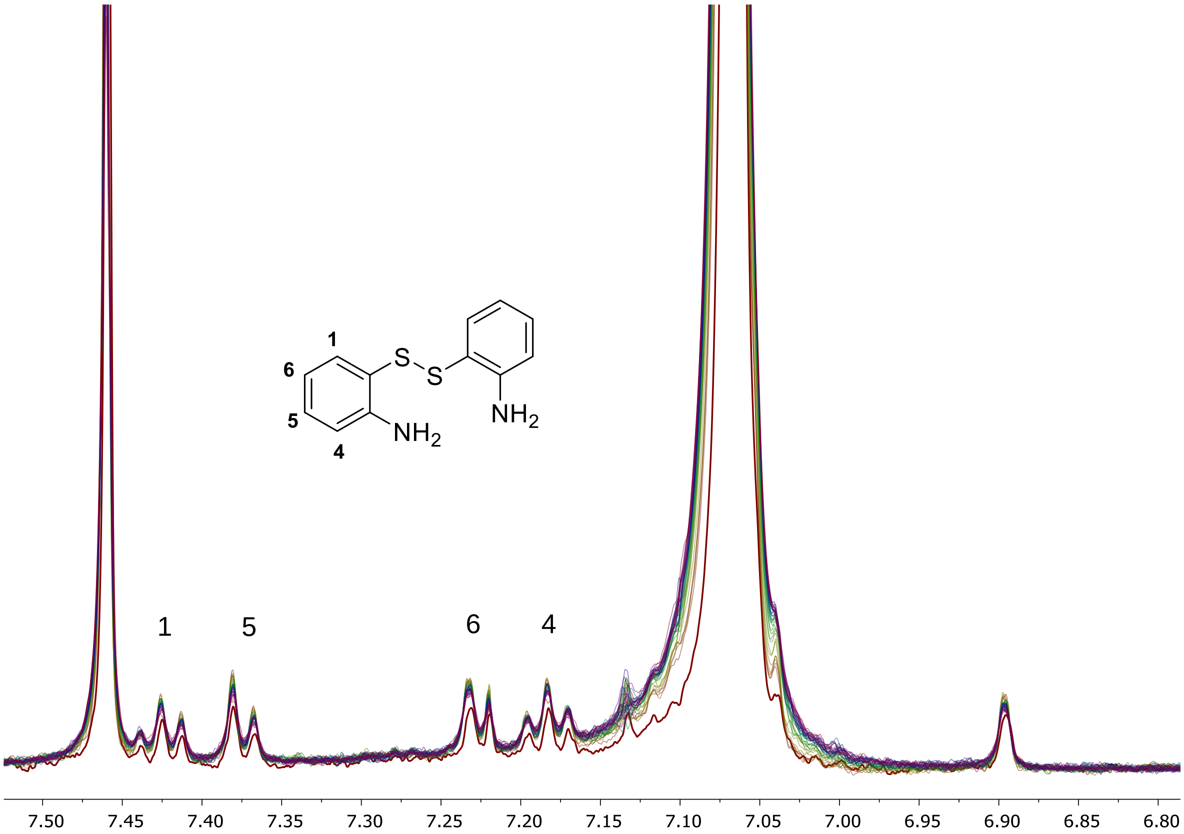

Figure S4. ^{1}H-NMR kinetic study to analyze the integral value during time, with overlapped data from different time points.

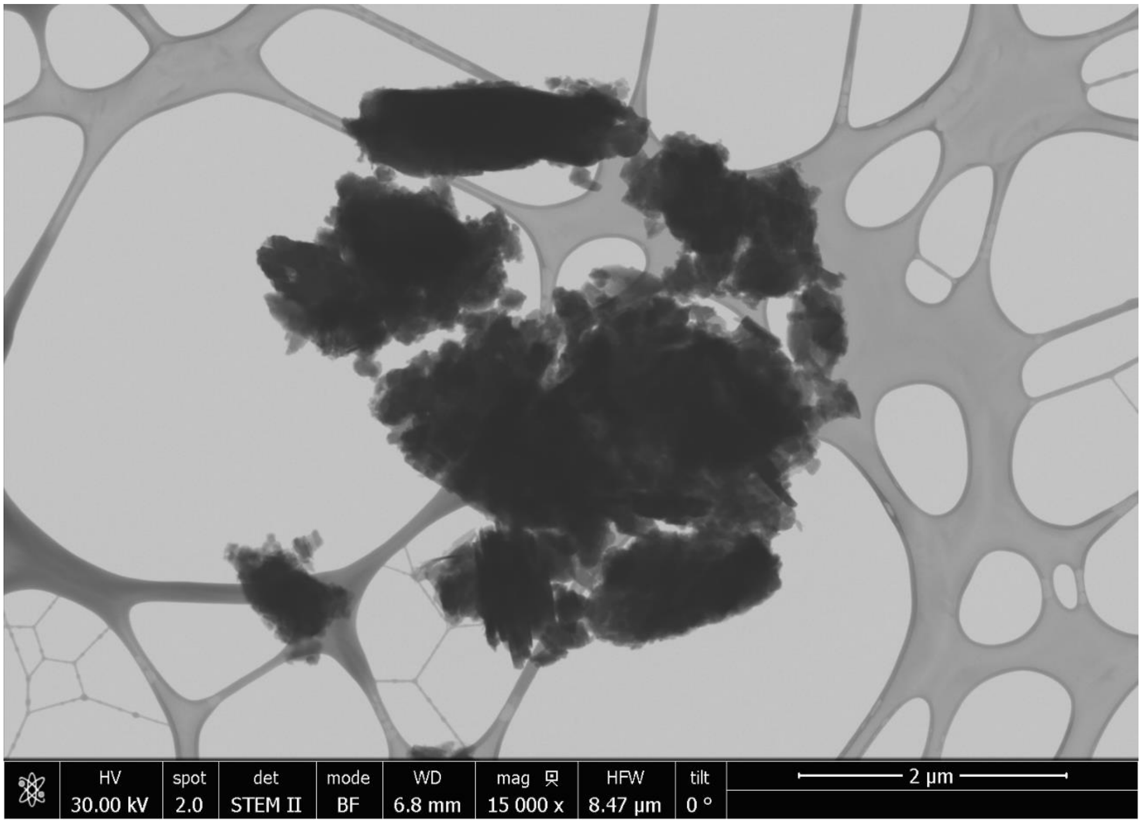

Figure S5. Degraded **MOF@DiS-NH$_2$** after nine moth of storage.

Table S1. HPLC method employed in calibration curve and quantification of **DiS-NH2**

**Time (min)**	**MeOH (%)**	**H_2O (%)**	**Flow (mL/min)**
0.0	75.0	25.0	1.00
15.0	75.0	25.0	1.00
16.0	100.0	0.0	1.00
20.0	100.0	0.0	1.00
22.0	75.0	25.0	1.00
25.0	75.0	25.0	1.00

Tables S2. HPLC method employed in calibration curve and quantification of **DiS-*O*-Acetyl**

Time (min)	MeOH (%)	H_2O (%)	Flow (mL/min)
0.0	65.0	35.0	1.00
15.0	65.0	35.0	1.00
16.0	100.0	0.0	1.00
20.0	100.0	0.0	1.00
22.0	65.0	35.0	1.00
25.0	65.0	35.0	1.00

Table S3. HPLC results of calibration curve to **DiS-NH$_2$**

Concentration (ppm)	Retention Time (min)	Area	Mean Area	S.D
300	4.753	1247283	1248497	8080
	4.74	1257116		
	4.733	1241093		
200	4.733	893470	881620	10969
	4.74	871821		
	4.747	879568		
100	4.74	416831	417035	9155
	4.74	407984		
	4.747	426291		
50	4.733	194977	196623	6893
	4.747	204190		
	4.733	190702		
25	4.733	111340	109561	1772
	4.733	107797		
	4.733	109547		
10	4.733	42715	40907	1843
	4.727	40976		
	4.72	39030		
5	4.74	19829	18804	1195
	4.733	17491		
	4.713	19092		
1	out of range			

Table S4. HPLC results of calibration curve to **DiS-*O*-Acetyl**

Concentration (ppm)	Retention Time (min)	Area	Mean Area	S.D
1000	10.253	8364882	8457856	83439
	10.24	8482460		
	10.247	8526227		
500	10.267	3983322	3937577	47498
	10.273	3888501		
	10.26	3940907		
200	10.273	1639626	1653165	24067
	10.28	1680953		
	10.267	1638917		
100	10.273	794043	786036	34647
	10.28	815979		
	10.253	748086		
50	10.267	438523	436529	16717
	10.267	452160		
	10.287	418905		
10	10.253	82185	82785	3469
	10.253	86514		
	10.3	79655		
5	out of range			
1	out of range			

Table S5. HPLC results of water solubility measurements of free bioactive compounds

Sample (ppm)	Injection	Area	Real Concentration after Calibration Curve (ppm)	Water Solubility (mg/L)
Dis-NH_2 (1000)	1	177959	36.01	36.47 ± 0.89
	2	185430	37.50	
	3	177498	35.91	
DiS-*O*-Acetyl (1000)	1	146572	20.42	19.47 ± 0.86
	2	134859	19.24	
	3	129922	18.75	

Table S6. HPLC results of encapsulation percentages inside the MOFs

Sample (ppm)	Injection	Area	Real Concentration after Calibration Curve (ppm)	Encapsulation (%)	Encapsulation Mean (%)
Dis-NH_2 (1000)	1	4211176	426.88	42.69	42.80 ± 0.10
	2	4228353	428.59	42.86	
	3	4226953	428.45	42.85	
DiS-*O*-Acetyl (1000)	1	1621114	167.87	16.79	16.71 ± 0.08
	2	1605627	166.32	16.63	
	3	1613515	167.11	16.71	
